# Primary School Children's Views and Habits Around School Lunches

**DOI:** 10.1002/hpja.70219

**Published:** 2026-07-21

**Authors:** Janandani Nanayakkara, Alison O. Booth, Claire Margerison, Anthony Worsley, Gozde Aydin

**Affiliations:** ^1^ Institute for Physical Activity and Nutrition, School of Exercise and Nutrition Sciences, Deakin University Geelong Australia; ^2^ School of Exercise and Nutrition Sciences, Deakin University Geelong Australia; ^3^ Centre for Health Economics, Monash University Australia

**Keywords:** children, primary school, school lunch, school‐provided lunch program

## Abstract

**Issue Addressed:**

Australia does not have a school‐provided lunch program. Recent research has explored various groups' opinions on having such a program; however, children's perceptions have been under‐explored. This study explored the current practises and perceptions of primary school children in Victoria, Australia, regarding school lunches.

**Methods:**

An online survey conducted between late 2022 and mid‐2024 gathered grades 4–6 children's opinions regarding school lunches.

**Results:**

Most children (84%, of total *n* = 138) reported bringing a packed lunch from home every day to school. Most children agreed that they like to have quick‐to‐eat food (84%) and warm or freshly cooked food (72%) for school lunch and have food warming and cooling facilities in school (61%). Slightly over half of children (52%) agreed that the time allocated for eating is long enough to eat their lunch. The most enjoyable aspects of school lunchtime were: socialising with friends, eating food, and the chance to break from learning. Aspects of school lunchtime that children disliked were: not having enough time to eat food, food rules, the lack of variety in meals or running out of food to eat, and unpleasant social interactions. When asked about their willingness to have school‐provided lunches, 47% chose ‘yes’, and 45% chose ‘maybe’.

**Conclusions:**

Among this sample of primary school children, home‐packed lunches remain the main food source at school lunchtime. They indicated a desire for additional lunchtime facilities and showed a willingness to have school‐provided lunches.

**So What?:**

Greater consideration of children's perspectives could support policymakers and health promoters in identifying and developing targeted strategies to improve primary school lunches and the food environment.

## 1 Introduction

Proper nutrition is essential for supporting children's physical and cognitive development [1, 2], enhancing academic performance [3], and promoting overall well‐being. With children spending 6–7 h each day at school and consuming more than one‐third of their daily food intake while at school, the school setting plays a crucial role in their diet [4]. In Australia, most children bring food from home for lunch and other breaks to eat throughout the school day, and many schools also offer food for purchase through canteens [5, 6]. Unfortunately, a significant proportion of Australian children's energy intake at school comes from energy‐dense, nutrient‐poor foods, such as biscuits, packaged snacks, cereal bars, and sugary drinks [4, 7]. The availability of unhealthy options in school canteens, coupled with low compliance to healthy canteen policies and a preference for less nutritious foods among children, remains a concern [8].

Australian families have previously reported that they often face challenges in providing nutritious and convenient food for school meals that meet children's preferences [9, 10, 11]. These challenges stem from various factors, including the high cost of food, limited time to prepare school lunches, and concerns about food safety [9, 10, 11]. Additionally, parents have criticised the limited time children are given to eat their lunch at school, which further complicates efforts to ensure proper nutrition [12].

Given that current school lunch practises are far from optimal in Australia, introducing alternative lunch programs, such as school provided meals that have been implemented in many other countries, could be a potential solution [13]. The provision of universal school lunches has become a topic of public debate in Australia, with support from various politicians and political parties advocating for their implementation [14, 15]. A recent systematic review of universal free school meal programs found evidence from multiple countries indicating improvements in diet quality, food security, academic performance, school attendance, and reduced financial strain on families, especially in programs that provided free lunch [16]. To effectively design and implement such programmes, it is important to first conduct a needs assessment to understand the perspectives and preferences of all relevant stakeholders. A number of recent Australian studies have investigated parents' and other relevant stakeholders' views regarding school lunch provision [9, 17, 18]. However, it is crucial to involve children in decision‐making, as emphasised by the United Nations Convention on the Rights of the Child [19].

Despite this, research into children's perspectives on school lunch experiences and school‐provided lunch models remains limited. Only one recent Australian study has explored students' perceptions of a school‐provided lunch model, employing a story‐completion method where students were given a brief story stem and asked to complete a story about a hypothetical school‐provided lunch scenario [20]. However, this study was conducted in a single school situated in an area with high socioeconomic status, in South Australia—a state in Australia—which limits the generalisability of its findings. Additionally, children's perspectives were collected during the evaluation of a recently launched school lunch program in Tasmania (another state in Australia), conducted across 30 schools during 2022–2023, which built upon an earlier school lunch trial in 2020 [21]. However, this feedback was specific to the trial evaluation and did not comprehensively address children's broader perspectives on school lunch models. Each state in Australia has its own school system policies and procedures, necessitating gathering state‐specific data to inform state‐level school policies and interventions. Recent studies focusing on perceptions of primary school children in Victoria, Australia, regarding school lunches are scarce.

To address these gaps, our study aims to investigate perceptions of primary school children in the state of Victoria regarding school lunch provision and their current lunch practises, providing valuable insights to inform the design of future initiatives.

## 2 Methods

### 2.1 Study Design and Participants

An online survey was employed in this study. Children in grades 4, 5, and 6 (aged 10–12 years) in primary schools in Victoria were invited to complete the survey.

### 2.2 Survey Instrument

The questionnaire employed in the current study included demographic questions (completed by parents) and a series of closed and open‐ended questions about primary school children's current practises and perceptions regarding school lunches (completed by their children). Some questions were adapted from a previous survey of parents' perceptions of school lunches [9, 22]. Other questions were specifically created for this survey to capture children's perceptions of school lunches. The questionnaire was pretested with two primary school children. Minor modifications were made to the wording and question order to improve the survey clarity and flow. The first section of the questionnaire asked questions related to the frequency of bringing a packed lunch to school, having lunch orders (i.e., online food ordering system where parents or children order food in advance from a menu and the food is delivered to a specific location within the school on the nominated day), and buying lunch from school [options: everyday, most days (3–4 days/week), some days (1–2 days/week), rarely (1–2 days/month), never]. Those who indicated they buy food from school were asked to choose all the places where they buy food (options: school canteen/tuckshop, vending machine, other). Then, they were asked to indicate the places where they eat their school lunch (options: inside the classroom, outside the classroom, canteen, another place), and with whom they eat lunch (options: I eat alone, I eat with my classmates, I eat with students from other grades, I eat while playing with other students, other).

Next, they were presented with five statements related to school food rules (i.e., policies) and asked to indicate if their school has such a policy or not. Following this, children were given space to write if they knew of or had heard of any other food‐related rules in their school. Then, children were given space to list three foods that they usually eat for school lunch and three foods that they would love to have for school lunch. Two open‐ended questions prompted them to write responses on what they most like about school lunchtime (time they spend eating their lunch) and anything that they do not like about school lunchtime. They were also asked to indicate their agreement on nine statements related to school lunches, lunch duration and the school food environment. Lastly, 10 images of cooked meals were presented (Figure S1), and the children were asked if they would choose these meals if their schools were to provide them as school‐provided lunches to all children. Following this, they were asked if they would like their school to provide lunches like these to all children. Finally, they could suggest other foods their school could provide for everybody.

Demographic questions obtained information on the children's grade, gender, school type (Government, Catholic, Independent (Private), Other), school postcode and home postcode. School postcode was used to derive school location, postcodes of major cities as urban and all other postcodes as rural. Home postcodes were used to derive the household socioeconomic status (SES) by matching the postcode to SEIFA 2021 Index of Relative Socio‐economic Advantage and Disadvantage (IRSAD) decile rankings. IRSAD summarises information about the economic and social conditions of people and households within an area [23]. The IRSAD deciles resulting from this matching were grouped into five groups: deciles 1 and 2 (most disadvantaged), deciles 3 and 4, deciles 5 and 6, deciles 7 and 8, and deciles 9 and 10 (most advantaged).

### 2.3 Data Collection

Data collection was carried out from late 2022 to mid‐2024. The survey was advertised to parents of primary school children using paid and unpaid social media (Facebook) advertisements. The survey was also advertised on Deakin University's Institute for Physical Activity and Nutrition's social media accounts. Additionally, the survey link was emailed to 262 parents who expressed their interest in participating in research related to school food and nutrition in a previous survey that explored parents' perceptions of school lunches [9]. Also, an email was sent by the school administrative officer to the staff and higher degree by research students of the School of Exercise and Nutrition Sciences, Deakin University, inviting those with a primary school child to participate in the survey.

The survey was built on the Qualtrics platform. Once parents clicked the link in the survey advertisement, they were taken to the landing page of the survey, where they could read and download the plain language statement and indicate their consent for themselves and their child to participate in this study. Then, they responded to two screening questions to determine their child's eligibility (living in Victoria, Australia and studying in grades 4, 5, or 6 in a primary school in Victoria, Australia) to participate in this study. If they were eligible, parents were asked to invite their child to complete the next section (school lunch practises and perceptions) of the survey. To obtain child assent, at the start of this section, children were asked if they wished to answer some questions related to school lunches. If they selected ‘yes’, they could move to the questions related to school lunches and if they answered ‘no’, the survey was ended. Once the children had completed all these questions, they were asked to hand over the survey to their parents again for them to answer all the demographic questions.

This study was approved by the Ethics committee of the Faculty of Health, Deakin University HEAG_H 151_2022.

### 2.4 Data Analysis

Quantitative data were analysed using SPSS software (IBM Corp, 2022, version 29). Frequencies and percentages were calculated for all relevant variables. *χ* ^2^ tests were performed to explore the associations between categorical variables of students' practises and perceptions and their demographic characteristics (gender, grade, household SES, school type, and school location). A *p*‐value less than 0.01 was set as the level of significance.

Students' written responses to the questions on the foods that they usually eat and foods that they would love to have for school lunch were extracted onto an Excel spreadsheet. These food items were manually categorised into food groups considering their similarities. The written responses to what they most liked or did not like about their school lunchtimes were extracted from the Qualtrics platform and uploaded to NVivo software (Lumivero, 2023, version 14). A template analysis technique [24] was employed to analyse the data. The first author read the responses to be familiar with the data and generated priori codes (i.e., coding template). The coding proceeded using this template and existing codes were modified or deleted, and new codes were added as required. The resulting template (i.e., themes) along with the associated quotes are shown in the Results section.

## 3 Results

### 3.1 Demographic Characteristics of the Children

Out of 267 respondents who clicked the survey link, six did not provide their consent for their child to participate, 24 did not meet the eligibility criteria or did not answer the eligibility questions, and 31 children did not provide their assent to complete the survey. Accordingly, 206 proceeded to the survey questions. Out of these respondents, 138 completed the survey (67 did not complete the survey questions and one respondent included a postcode outside Victoria, hence was removed from the sample). The characteristics of these 138 respondents (i.e., primary school children) are shown in Table 1.

**TABLE 1:** Demographic characteristics of children (*N* = 138).

Children characteristics	*n* (%)
Gender	
Male	69 (50)
Female	67 (49)
Gender diverse	1 (1)
Prefer not to say	1 (1)
Grade	
4	59 (43)
5	44 (32)
6	35 (25)
Household Socio‐economic status^a^	
Deciles 1 and 2 (Most‐disadvantaged)	12 (9)
Deciles 3 and 4	14 (10)
Deciles 5 and 6	23 (17)
Deciles 7 and 8	39 (29)
Deciles 9 and 10 (Most‐advantaged)	49 (36)
Missing	1
School type	
Government	93 (67)
Non‐government^b^	45 (33)
School location	
Urban	102 (75)
Rural	34 (25)
Missing	2

^a^ Based on IRSAD (relative socio‐economic advantage and disadvantage) deciles.

^b^ Included catholic and independent schools.

Males and females were equally distributed in the sample. Nearly half of the children (43%) were studying in grade 4 and the rest were from Grades 5 and 6. Most children were studying in government schools (67%) and schools located in urban areas (75%). The majority of children (65%) resided in areas of high socio‐economic advantage, as indicated by IRSAD deciles 7–10 (Table 1).

### 3.2 Students' School Lunch‐Related Behaviours and Practises

Most children (84%) reported that they brought a packed lunch from home every day to eat during school (Table 2). Slightly less than 50% of children reported that they rarely (1–2 days/month) had lunch orders, whilst nearly one‐third (32%) reported never having lunch orders. When asked about buying lunch at school, most children (76%) chose ‘never’ whilst 20% chose rarely (1–2 days/month). Children who indicated that they buy lunch at school were asked to select all the places where they buy food; the most chosen option was ‘school canteen/tuckshop (85%) (Table 2). Most children (87%) reported that they ate their school lunch ‘inside the classroom’ while ‘outside the classroom’ was chosen by 73% of children (children could select all the places applicable to them). More females (85%) compared to males (62%) chose ‘outside the classroom’ (*χ* ^2^ = 9.044, *p* = 0 0.003). Finally, they were asked to select the people they ate their lunch with, 91% chose ‘I eat with my classmates' whilst slightly over one‐fifth of children chose ‘I eat with children from other grades' (26%) and ‘I eat while playing with other children’ (24%). Only a small percentage of children (7%) chose ‘I eat alone’ (Table 2). There were no other significant associations between these behaviours and practises and the demographic characteristics of the children (gender, grade, household SES, school location, and school type).

**TABLE 2:** Primary school lunch related behaviours and practises (*N* = 138).

School lunch related behaviours and practises	*n* (%)
Frequency of bringing a packed lunch from home	
Everyday	116 (84)
Most days (3–4 days a week)	21 (15)
Some days (1–2 days a week)	1 (1)
Frequency of having lunch orders	
Some days (1–2 days a week)	27 (20)
Rarely (1–2 days a month)	67 (49)
Never	44 (32)
Frequency of buying lunch at school	
Everyday	1 (1)
Some days (1–2 days a week)	4 (3)
Rarely (1–2 days a month)	28 (20)
Never	105 (76)
Places where food was brought at school^a^	
School canteen/tuckshop	28 (85)
Vending machine	2 (6)
Other^b^	5 (15)
Places where school lunch was eaten^a^	
Inside the classroom	120 (87)
Outside the classroom	101 (73)
Canteen	4 (3)
Another place^c^	10 (7)
People children eat lunch with^a^	
I eat with my classmates	126 (91)
I eat with children from other grades	36 (26)
I eat while playing with other children	33 (24)
Other^d^	7 (5)
I eat alone	10 (7)

^a^ Students could choose more than one option.

^b^ Written responses included fundraising events, lunch order from school supplier, order online, and school orders from outside.

^c^ Written responses included a few different places on school premises such as a courtyard, covered area, gym, eating area, and playground.

^d^ Written responses included with my friends, my friends from other classes in the same grade, the whole school eats together in the eating area, my class and teacher.

### 3.3 Usual School Lunch Food and Preferred School Lunch Food

**FIGURE 1:**
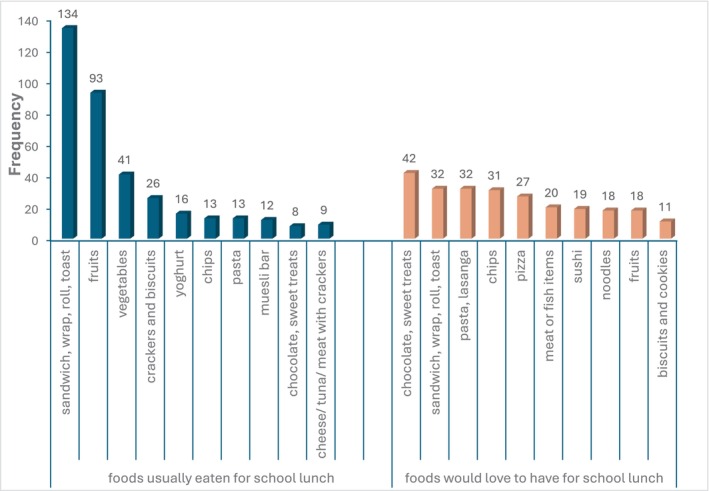
Foods primary school children usually eat for school lunch and foods they would love to have for school lunch.

The Figure 1 summarises the most frequently reported foods that children usually consume for their lunch and foods that they would love to have for their lunch. When considering the foods usually eaten for school lunch, ‘sandwich, wrap, roll, toast’, fruits, vegetables, ‘crackers and biscuits’, and yoghurt were the most commonly reported food items. Except for ‘sandwich, wrap, roll, toast’, none of these food items were in the top five list of foods that they would love to eat. Chocolates and sweet treats were the most frequently reported food on the ‘would love to eat’ list but not on the ‘usually eaten’ list.

### 3.4 School Food Rules

Very few children were unaware of school food rules (Table 3). Most children (86%) reported they are not allowed to share their food with their friends/classmates. Slightly over half of children (54%) reported they are not allowed to bring nuts to school. Less than 20% of children reported they are not allowed to bring eggs (12%) and food wrapped in food wrappers (17%). Finally, 21% reported that they are not allowed to bring lollies, candy, or chocolates to school (Table 3). There were no significant associations between children's perceptions of school food rules and their demographic characteristics.

**TABLE 3:** Children's perceptions of school food rules and their agreement with statements related to school lunches, lunch duration and food environment (*N* = 138).

Food rules		*n* (%)
Are you allowed to share food with your friends/classmates?	Yes	14 (10)
	No	119 (86)
	I don't know	5 (4)
Are you allowed to bring nuts to school?^a^	Yes	56 (41)
	No	74 (54)
	I don't know	7 (5)
Are you allowed to bring eggs to schools?	Yes	98 (71)
	No	17 (12)
	I don't know	23 (17)
Are you allowed to bring food wrapped in food wrappers to school?^a^	Yes	109 (80)
	No	23 (17)
	I don't know	5 (4)
Are you allowed to bring lollies, candy, or chocolates to school?	Yes	101 (73)
	No	29 (21)
	I don't know	8 (6)

Statements related school lunches, lunch duration and food environment		
I like to eat my lunch first and then play at school.	Yes	119 (86)
	No	19 (14)
I like to have quick‐to‐eat food (e.g., sandwiches) for my school lunch.	Yes	116 (84)
	No	22 (16)
I like to eat warm or freshly cooked food for my school lunch.^a^	Yes	99 (72)
	No	38 (28)
If my friend brought food that looked good or smelled good, I ask mum or dad for that food in my lunch.	Yes	93 (67)
	No	45 (33)
I like to eat my lunch outside of the classroom.	Yes	93 (67)
	No	45 (33)
I like to have food warming (e.g., microwave) and food cooling (e.g., fridge) facilities at my school.^a^	Yes	84 (61)
	No	53 (39)
Time given for eating is long enough to eat all my lunch if I want to.^a^	Yes	72 (52)
	No	65 (47)
I help make my school lunch.^a^	Yes	69 (50)
	No	68 (50)
I like to eat my lunch while playing.	Yes	44 (32)
	No	94 (68)

^a^ *n* = 137, one missing.

Children's written responses related to other food‐related rules included food safety related rules (e.g., *‘If you have someone in your class who is allergic to a food you can't have it’*), rules to encourage healthy eating (e.g., ‘*bring a brain food (fruit or veggies)* *’*, ‘*No fast food and no soft drink*’), *f*ood waste related policies (e.g., *‘Encouraged to eat food without wrapping on Wednesday. Our school calls it ‘wasteless Wednesday’*), and rules to control the eating behaviours (e.g., *‘Have to stay at the eating area for first 10 min of lunch’, ‘Only eat in the eating area’*).

### 3.5 Children's Agreement With Statements Related to School Lunches, Lunch Duration and Food Environment

More than 80% of children liked to eat their lunch first before playing and to have quick‐to‐eat food for their school lunch, whilst 72% liked to eat warm or freshly cooked food for their lunch. Two‐thirds of children liked to have their lunch outside and to have food warming and cooling facilities at school. Approximately half of the children agreed that the time allowed for eating is adequate and a similar proportion reported they help make their school lunch. Only one‐third of children liked to have their lunch while playing (Table 3).

More non‐government school children (64%) disagreed with the statement *‘*time given for eating is long enough to eat all my lunch if I want to*’*, compared to government school children (40%) (*χ* ^2^ = 6.815, *p* = 0.009). There were no other significant associations between children's perceptions and their demographic characteristics.

### 3.6 What Do Children Most Like About School Lunchtime?

All the children responded to the question ‘What do you most like about school lunchtime (think only about the time you spend eating, not the time spent playing)?’, and most of these responses were limited to a few words. Three major themes were derived from their responses.

#### 3.6.1 Theme 1: A Socialising Opportunity

Socialising opportunities were mentioned 66 times, and these opportunities include eating with friends (e.g., ‘*You eat lunch with your friends’*), talking with friends (e.g., ‘*enjoy being with friends and chatting’*), and talking with friends while eating (e.g., ‘*I guess I like sitting with my friends and chatting while we eat our lunch’*).

#### 3.6.2 Theme 2: Food‐Related Aspects

Food‐related aspects were mentioned 28 times. Some children mentioned specific features of their lunch such as *‘yummy food’, ‘mum makes good lunch*. *It is healthy and I get to pick my snack’*, and *‘* *I get* *to eat favourite foo*d*’*, while some others alluded to the physiological benefits of lunch intake such as ‘*not feeling hungry* *’* and *‘* *filling my tummy* *’*. Some children referred to the anticipation of finding out what they have for lunch as expressed by the following quotes: ‘*finding out what yummy food my mum has packed*’ and *‘* *surprises in my lunchbox* *’*.

#### 3.6.3 Theme 3: Break From Learning

Lunchtime as an opportunity to get a break from learning was mentioned seven times, two example quotations are *‘* *I don't have to do school work when we are eating* *’*, and *‘* *break from school work* *’*.

### 3.7 What Do Children Dislike About School Lunchtime?

All the children answered the question ‘Is there anything that you don't like about school lunchtime?’ Their responses were categorised into four themes.

#### 3.7.1 Theme 1: Time‐Related Aspects

Time‐related aspects of the school lunchtime were mentioned 51 times. Some of the responses (25 mentions) were specifically related to the time allocated or available to eat school lunch and children voiced their concerns regarding the inadequacy of time as follows; *‘* *not getting enough time to eat* *’*, *‘* *rushing because we only get ten minutes* *’*, and ‘*too quick and I don't get to finish my food*’. A further 24 responses referred to the inadequacy of school lunchtime in general (i.e., did not include the term eating or food in their responses) (e.g., ‘*It's not long enough*’, ‘*It goes too fast’*). There were two responses regarding the start time of school lunch, one was about early lunch, ‘*it's too early‐11.30 lunch* *’* whilst the other response was about late start time, *‘* *too late in day* *’*.

#### 3.7.2 Theme 2: The Food Environment and Lunchtime Rules

There were 21 mentions regarding the school lunchtime rules and food environment.

Different lunchtime rules (18 mentions) were mentioned. These included not being allowed to go outside or move around until students finish eating (e.g., ‘*I'm not allowed to move around when I'm finished unless I'm putting my lunch away*’, *‘we can't play until we've eaten lunch’*), not allowing to have interactions with others (e.g., ‘*no sharing’*, ‘*We don't eat with other classes’*), having to be quiet while eating (e.g., ‘*If we can't talk it's boring*’), and having to sit at a specific place to eat their lunch (e.g., *‘having a set spot to sit’*). There were three mentions of dissatisfaction regarding the school food environment, *‘* *not having enough seating* *’*, *‘* *mess in the yard* *’*, and *‘no tuckshop’*.

#### 3.7.3 Theme 3: Food Characteristics

Perceptions related specifically to the food consumed at lunchtime were mentioned 14 times.

Most of these were regarding their own food (10 mentions), and these were related to lack of variety (e.g., *‘Having the same food everyday’*), ‘*running out of food in lunchbox*’ and food being cold (e.g., *‘Sometimes the food is cold’*). There were two contradicting opinions related to others' lunches as expressed by the following quotes, ‘*Some of the other* *kids’* *food smells yuck’* and ‘*I get jealous of other people's lunch*’.

#### 3.7.4 Theme 4: Unpleasant Social Interactions

Unpleasant social interactions were mentioned five times which included fights with friends (e.g., *‘When there are fights with friends’*), friends being mean (e.g., *‘If my friends are being mean’*), and bullying.

### 3.8 Possible School‐Provided Lunches

Children were presented with 10 different meal photos with their names and asked if they would choose these meals if their schools were to provide these as lunches for all the children. Most children (84%) chose ‘spaghetti bolognese’, and ‘a wrap with chicken and vegetables’ (75%). The next most chosen meals were ‘rice and chicken curry’ (65%) and ‘roast chicken with vegetables’ (60%). The meals that were chosen by less than 50% of the children included ‘vegetable pizza’ (46%), ‘pasta with tuna’ (40%), ‘pumpkin soup’ (37%), and ‘vegetable salad’ (35%). The least popular options were ‘baked potatoes with tuna’ (26%) and ‘rice with chickpea/lentil curry’ (22%). There were no significant associations between children's preferences for meals and their demographic characteristics.

When asked if they would like their school to provide lunches like these to all children, 47% chose ‘yes’, 45% chose ‘maybe’, and 8% chose ‘no’. There were no associations between willingness to have school‐provided lunches and children's demographic characteristics.

When asked what other food the school could provide for school lunch for everybody, the most commonly mentioned food items were sushi (25 times), sandwiches, rolls, and toast (23 times), and pizza (17 times). The other foods that were mentioned more than 10 times each included chips, fruit, and pies. Pasta, rice, chicken, and hot dog were mentioned between 5 and 9 times. Several other food items were mentioned less frequently (Table S1).

## 4 Discussion

This survey explored perceptions and practises of primary school children in Victoria, Australia, regarding school lunches. The findings show that a home‐packed lunch was by far the most common source of food during school lunches and there were differences between what is commonly consumed for school lunches and what children would prefer to eat for school lunches. Children's responses show their expectations regarding school lunches and the food environment, including the availability of warm or freshly cooked food, the availability of food warming and cooling facilities in the school, and increasing the time allocated to eating lunch. The findings also show children's willingness to have school‐provided lunches and their preferences for different food items in such meals.

Most children in this sample reported bringing a packed lunch from home every day to school. This finding matches earlier Australian studies [4, 5, 9], highlighting that home‐packed lunches remain important contributors to children's food intake during school hours. The top five foods usually consumed at lunchtime were sandwiches, fruits, vegetables, crackers and biscuits, and yoghurt indicating the presence of both core and discretionary food in school lunches. This is also consistent with earlier studies. For example, in a recent Australian survey, a substantial proportion of parents reported providing some core foods every day in home‐packed lunches [fruits (94%), vegetables (57%) and sandwiches (54%)], whilst discretionary foods like salty crackers and sweet cookies/biscuits were provided 3–4 times/week by 50% and 40% parents, respectively [9]. A recent audit of Australian school lunches also found bread and fruits as common core food items in primary school lunches [25]. This audit further reported that ‘extras’ (i.e., discretionary food items) were also common in school lunches.

Children's responses regarding their preferred school lunch food items indicate a discrepancy between their current school lunch consumption and their expectations or desires. While sandwiches were desired and consumed, foods such as chocolates and sweets, pasta and lasagna, chips and pizza were desired but not consumed. This may be due to the unavailability of food warming facilities to reheat some of these foods, as well as parental preferences. Unfortunately, the preferred food items mainly feature discretionary food items, which according to national guidelines [26], should only be consumed occasionally and should not be consumed every day. Further studies should explore the factors that may influence children's preferences for core and discretionary food at school (e.g., knowledge, advertising, convenience, peer influence) to determine the strongest predictors. Such evidence would be crucial in developing interventions that target key influential factors of children's food preferences, helping shift preferences away from discretionary food and towards healthier core foods. For example, using peer modelling to increase preference for and consumption of healthier core foods.

The presence of a range of school food rules was apparent in children's responses, for example, ‘not allowing to share food or bring certain food to school’, rules that promote healthy eating or discourage unhealthy foods, and rules to reduce food waste, and non‐uniform implementation of such rules across primary schools in Victoria. This confirms previous research related to school food rules and policies which have also reported the presence of varying food rules and their inconsistent implementation across schools [9, 27, 28]. Our intention was to capture children's perceptions of school‐stipulated rules. However, it is possible that some of the food rules enforced by their family (e.g., no sweets at schools) may also have been considered school food rules.

Our current study provides further insights into children's perceptions of some food rules. In particular, the qualitative responses indicate their displeasure regarding rules related to mealtime behaviours, such as not allowing them to talk while eating, not allowing them to share food, and not allowing them to move around or play before finishing eating. Such food rules/policies help to maintain discipline during the lunch period and reduce the chance of food contamination and cross‐contamination. However, many of the children valued school lunchtime as an opportunity to socialise with friends. Studies from other countries with similar food practises (e.g., Norway—home‐packed lunches) [29] and different school meal environments (e.g., Sweden—school‐provided lunches) [30, 31] have also found children engage with their peers during mealtimes and both children's and their caregivers' appreciation of such interactions. Social interactions during mealtimes are also acknowledged in some dietary guidelines, such as Canadian [32] and Brazilian dietary guidelines [33], based on evidence of the benefits of such interactions on positive eating habits. This suggests that any restrictions in social interactions during lunchtime should be evaluated for their potential impact. Such evaluations could be used to guide the development of rules that help foster a positive social environment during school lunchtime.

Our results offer crucial evidence for children's perceptions about the time allocated to eating lunches. Children's disagreement (47%) with the statement ‘time given for eating is long enough to eat all my lunch if I want to’ along with their written responses related to the inadequacy of time given to eating, emphasised that not enough time is allocated for them to eat and enjoy their lunch at school. Our findings corroborate previous survey findings, where parents and teachers have also expressed concerns related to the inadequacy of time given to eating at school [9, 12]. We did not ask children how much time is allocated to eating. However, a recent study reported that most Australian primary school children get 10 min to eat their lunches [12]. Together, these findings suggest the need for extending the duration of school lunch time. Further studies should explore the most appropriate time duration for lunch consumption (i.e., 15 or 20 min) and the ways to increase the time allocated for eating with minimal disruptions to play and existing academic programs. An ideal starting time for lunch should also be established as schools seem to have varying lunch schedules and there are no guidelines for schools to follow for setting a lunch time.

Children's agreement with statements such as ‘I like to eat warm or freshly cooked food for my school lunch (72%) and ‘I like to have food warming (e.g., microwave) and food cooling (e.g., fridge) facilities at my school’ (61%) indicates their preference for warm and fresh meals for their school lunch. Most Australian primary school children do not have the opportunity to eat warm, freshly cooked food for lunch, as they typically consume home‐packed lunches during lunchtime. Food warming and cooling facilities tend to be non‐existent in this setting, especially in primary schools. Provision of food cooling and warming facilities would help them store food in the fridge and reheat at lunchtime, if required. This could reduce food safety concerns during the warmer months, potentially improving the variety of food in packed lunches. Also, this would facilitate students to enjoy warm (i.e., reheated) meals. However, the feasibility of providing such facilities remains to be investigated. Universal school‐provided meals would be one way to enable all primary school children to eat warm and healthy meals at school lunchtime.

Finally, the findings underscore children's willingness to have school‐provided lunches. It is interesting to note that children's agreement when they were asked, if they would like their school to provide lunches (47% and 45% of children chose ‘yes’ and ‘may be’ respectively) is similar to findings from other research which found both parents' and teachers' reported positive perceptions of school‐provided meals; 57% and 34% of parents chose ‘yes’ and ‘maybe’, respectively, when asked if they would let their child consume school‐provided lunches [22], and 29% and 46% of teachers chose ‘yes’ and ‘maybe/unsure’, respectively, when asked if primary schools should have a school‐provided lunch program [34].

Children's preferences for different food items from options provided showed that they prefer meals that have a combination of meat or poultry with a carbohydrate source such as wraps, spaghetti, and rice. Although for a previous question in this survey children expressed a preference for various discretionary foods for their school lunch, their choices regarding school‐provided lunches show a contrasting pattern. These findings suggest that when children are offered healthy school‐provided lunches, they are willing to consume them. This presents an important opportunity to improve their preferences for healthier foods and establish healthier eating habits. However, meal items with vegetables (e.g., vegetable pizza, vegetable salad), and soup (pumpkin soup) seem to be the least favoured food items. The lunch trial evaluation in Tasmania (a state in Australia) found a similar preference trend [35]. In a previous survey [22] parents suggested that school‐provided lunches should be healthy and should include more vegetables. Given that most Australian children do not meet the recommended intakes for vegetables [36], it is important to explore ways of increasing the vegetable content of school meals in ways that are appealing to children.

The findings have three main implications for local research and practise. First, it is important to explore ways of improving children's preferences for healthier core food options to be included in school lunches. Second, school leaders and policymakers should consider if it is feasible to provide food warming and cooling facilities for school children and ensuring adequate time is given to eat and enjoy lunch. Lastly, it is important to implement and evaluate a trial school‐provided lunch program in Victoria and elsewhere. The findings have broader implications for countries similar to Australia that do not have an established school‐provided lunch program and are considering transitioning to such lunch models. The children's perceptions we reported in this study could be used as a basis for creating survey or interview questions to explore country‐specific children's perceptions to help inform the designing of their lunch programs.

To the best of our knowledge, no other recent studies have explored perceptions of primary school children in Victoria regarding school lunches and school‐provided lunches. Their responses to open‐ended questions enabled us to obtain some insights into their perceptions of school lunches. However, their qualitative written responses were quite short. A future qualitative interview study would provide a more detailed understanding of themes identified in the present study.

It is important to acknowledge the limitations associated with the small sample size. This limited our ability to see any potential demographic differences in children's perceptions. We recruited children studying in grades four to six. Future studies could include all primary school children. However, the survey questionnaire would have to be tailored to suit the different age groups. Our sample was composed of a high proportion of children from the most advantaged households and schools in urban areas. Also, we do not know how many schools were represented by these 138 children; however, 97 different school postcodes were reported. Therefore, it is reasonable to assume that these children were studying at various schools across Victoria. However, the findings should be interpreted with caution as they may not be generalisable to all primary school children in Victoria.

## 5 Conclusions

This study shows that in this sample of primary school children in Victoria, home‐packed lunches remain the main food source at school lunchtime, and these lunches include both core and discretionary foods. Children's responses suggest a desire for additional facilities like food warming and cooling facilities at school, relaxation of certain food rules, and the allocation of more time to eat lunch. Their willingness to have school‐provided lunches supports the idea of implementing a universal school meal program for all primary schools in Victoria.

## Author Contributions

**Janandani Nanayakkara**, **Alison O. Booth**, **Claire Margerison**, **Anthony Worsley**, and **Gozde Aydin:** conceptualization. **Janandani Nanayakkara**, **Alison O. Booth**, **Claire Margerison**, **Anthony Worsley**, and **Gozde Aydin:** methodology. **Janandani Nanayakkara:** formal Analysis. **Janandani Nanayakkara:** writing original draft (abstract, methods, results and discussion). **Gozde Aydin:** writing original draft (introduction). **Alison O. Booth**, **Anthony Worsley**, **Claire Margerison:** writing – review and editing. All the authors read and approved the final manuscript.

## Funding

This study was supported by an internal grant from the Institute for Physical Activity and Nutrition Sciences, Deakin University.

## Ethics Statement

This study was approved by the Ethics committee of the Faculty of Health, Deakin University [HEAG‐H 151_2022]. Once parents clicked the link in the survey advertisement, they were taken to the landing page of the survey, where they could read and download the plain language statement and indicate their consent for themselves and their child to participate in this study. Then, they responded to two screening questions to determine their child's eligibility (living in Victoria, Australia and studying in grades 4, 5, or 6 in a primary school in Victoria, Australia) to participate in this study. If they were eligible, parents were asked to invite their child to complete the next section (school lunch practises and perceptions) of the survey. To obtain child assent, at the start of this section, children were asked if they wished to answer some questions related to school lunches. If they selected ‘yes’, they could move to the questions related to school lunches and if they answered ‘no’, the survey was ended.

## Conflicts of Interest

The authors declare no conflicts of interest.

## Supporting information


**Figure S1:** Images of school meals.

**Table S1:** Food items children prefer to have for school‐provided lunches.

## Data Availability

The authors do not have permission to share data.
