# Supplementary figures and images for: Primary School Children's Views and Habits Around School Lunches
Source: Health Promot J Austr. 2026 Jul 21;37(4):e70219. doi: 10.1002/hpja.70219 (PMC13386181; doi:10.1002/hpja.70219)

**Supplementary Figure 1: Images of school meals**


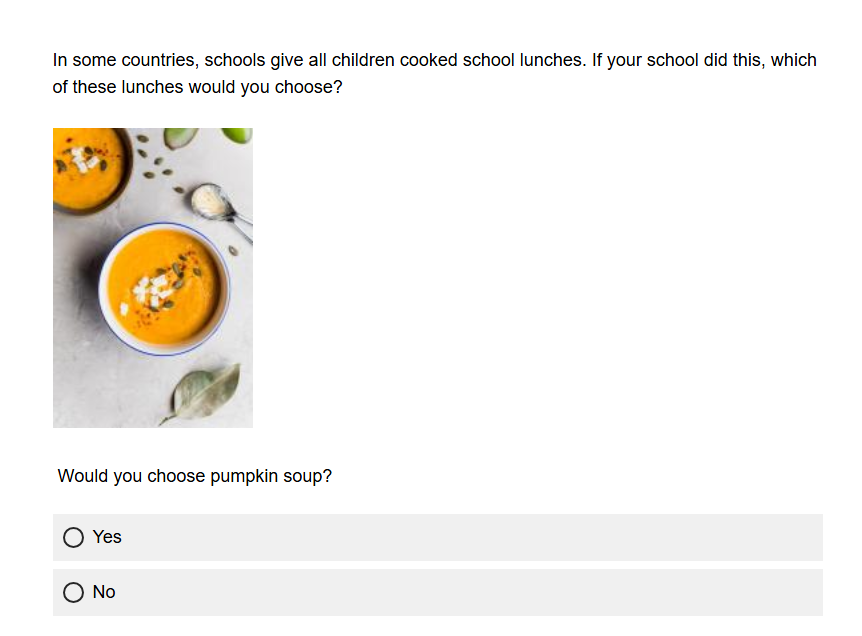


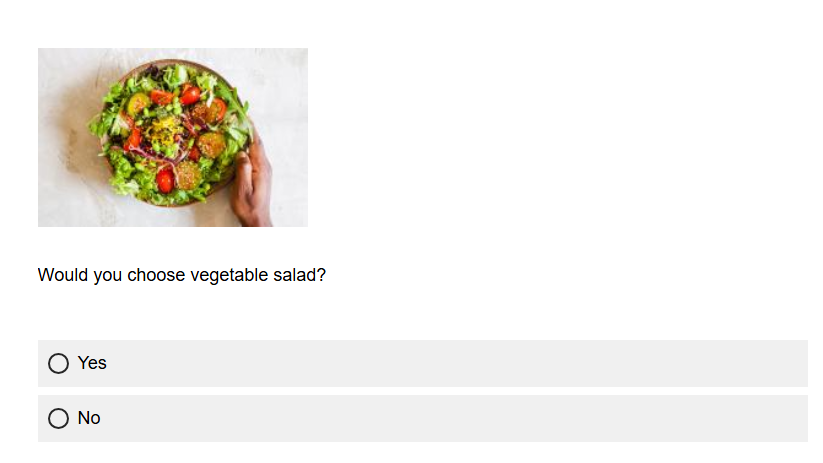


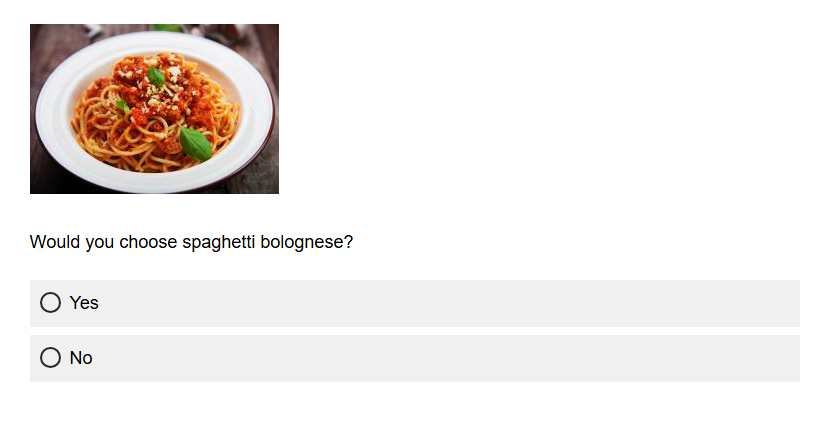


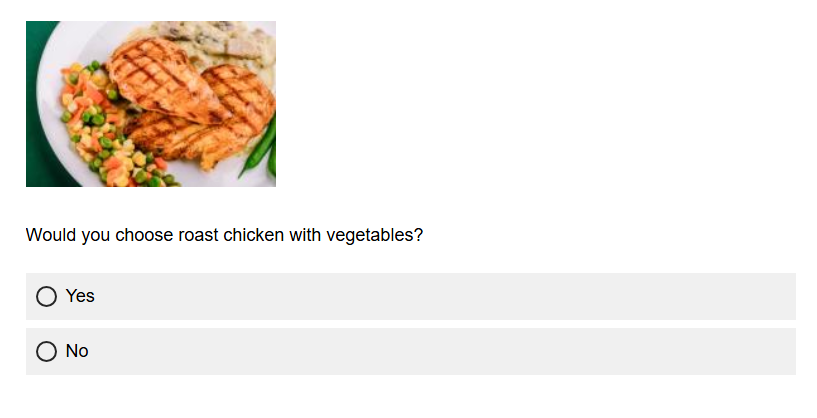


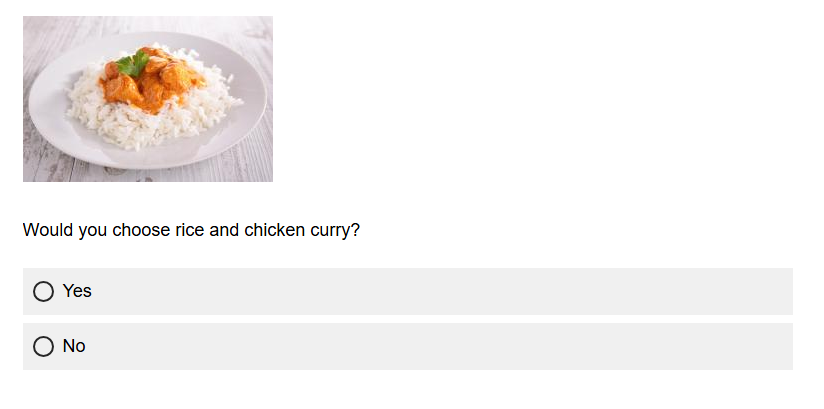


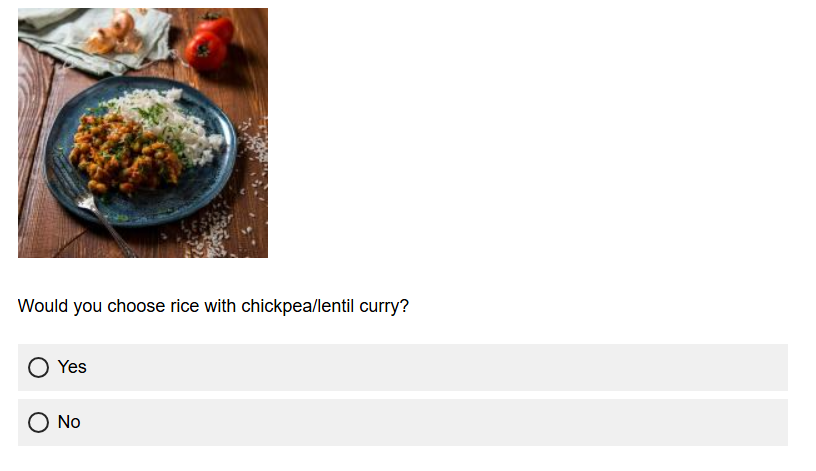


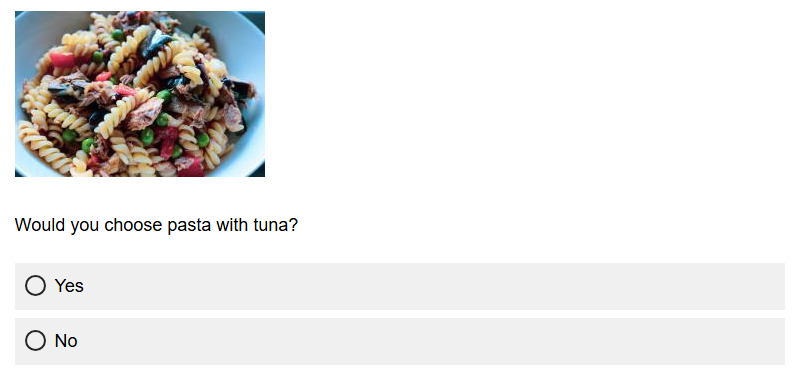


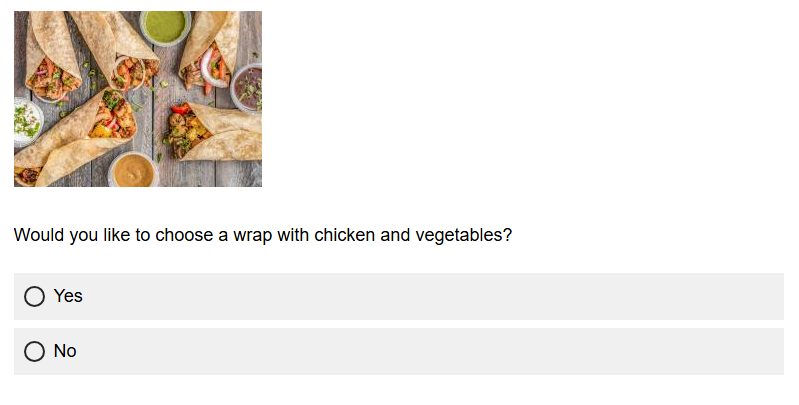


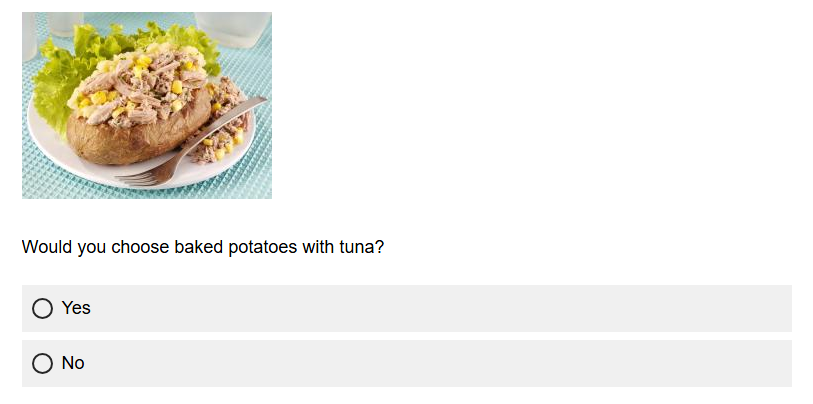


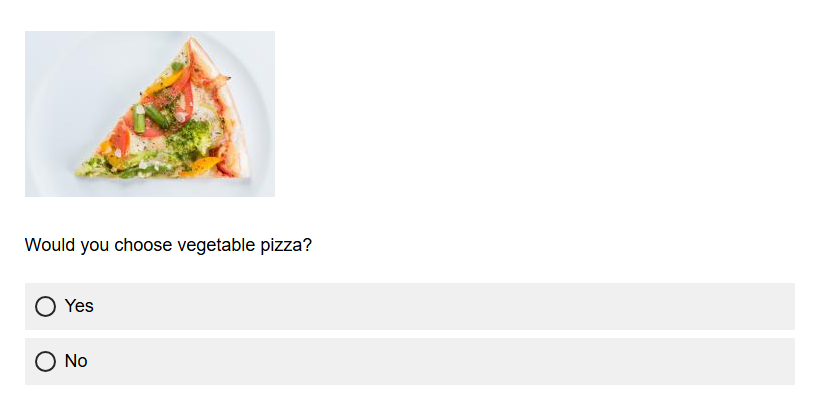

Supplement: Supplementary file 1 — Figure S1: Images of school meals. [file HPJA-37-0-s002.docx]
