# Supplementary material for: Primary School Children's Views and Habits Around School Lunches
Source: Health Promot J Austr. 2026 Jul 21;37(4):e70219. doi: 10.1002/hpja.70219 (PMC13386181; doi:10.1002/hpja.70219)
Supplement: Supplementary file 2 — Table S1: Food items children prefer to have for school‐provided lunches. [file HPJA-37-0-s001.docx]

**Supplementary Table I: Food items children prefer to have for school-provided lunches**

|  | Variations | Frequency |
| --- | --- | --- |
| Sushi | - | 25 |
| Sandwiches, rolls and toast | toasted sandwiches, different sandwiches, make your own chicken wrap, make your own sandwiches, salami sandwiches, salad rolls, sausage rolls | 23 |
| Pizza | margherita pizza, meat pizza, ham pizza, pizza without vegetables, healthy pizza | 17 |
| Chips | hot chips, fish and chips | 14 |
| Fruit | fruit platter, fruit salad | 13 |
| Pie | meat pie, spinach pie, party pie, shepherd’s pie | 13 |
| Pasta | pesto pasta, basil pesto pasta with chicken | 9 |
| Rice | fried rice, fried chicken rice, fried vegetable rice, chicken and rice, Japanese curry with rice | 9 |
| Chicken | honey chicken, roast chicken with gravy, roast meat with vegetables, schnitzels, satay sticks | 6 |
| Hot dog | - | 5 |
| Soup | pumpkin soup, soup with chunky vegetables, vegetable soup | 4 |
| Vegetables | Caesar salad, salads, veggie sticks with dip | 4 |
| Dumplings | - | 4 |
| Noodles | gluten free noodles, Singapore noodles, stir fry noodles | 4 |
| Chicken nuggets |  | 3 |
| Fast food | fast food, (name removed_1), (name removed_2) | 3 |
| Drinks | milk shake, hot chocolate | 3 |
| Hot food | - | 3 |
| Nachos | - | 3 |
| Tacos | - | 3 |
| Curries | Indian/Thai curries, potato curry | 2 |
| Tuna | tuna bake, tuna dip | 2 |
| Dim sims | - | 2 |
| Donuts | - | 2 |
| ice cream, icy poles |  | 2 |
| Eggs | eggs (fried, boiled, scrambled, or poached), omelette | 2 |
| Desert |  | 2 |
| Wrap |  | 2 |
| Quiche |  | 2 |
| Yoghurt | yoghurt with berries | 2 |
| Rice paper rolls |  | 2 |
